# A System of Surgery

**Published:** 1913-03

**Authors:** 


**A System of Surgery.** Edited by C. C. CHOYCE, B.Sc., M.D., F.R.C.S. Vol. II. Pp. xii, 1105. London: Cassell & Co., Ltd. 1912. 21s. net.

Last year we had the opportunity of reviewing the first volume of this work, with which we were very favourably impressed. Although the present volume deals largely with branches of surgery in which few striking advances have been made during quite recent years, and although, as a necessary consequence, the main bulk of its material has already appeared in other modern text-books, yet there will be found herein embodied a great deal of recent research work and discovery. Recent advances in anatomy, physiology, embryology, pathology and other branches of science have been drawn upon wherever they tend to throw light upon the problems of surgery proper.

This volume is largely occupied with the surgery of the abdomen and alimentary tract. Each section is treated fully, though without unnecessary elaboration. Only the more essential operations, and those best in accord with our present-day knowledge are described, and there is a complete absence of those obsolete methods which survive, alas! in too many would-be authoritative works.

Sampson Handley contributes an article on the breast, and includes in it the results of his important researches concerning the spread of carcinoma. He also sets forth an ingenious theory as to the causation of "Paget's disease of the nipple." He attributes this chronic eczematous condition to interference with the drainage of lymph from the nipple and areola. He postulates the presence, in every case, of a carcinoma in the breast which, having permeated the mammary lymphatics, has led to interference with the drainage of lymph from the subareolar plexus.

Several of the writers speak highly of the beneficial effects of a prophylactic course of X-rays after operations for the removal of cancer. Statistics are given in support of this procedure.

Urinary surgery is dealt with by Thomson Walker, and that of the male and female genital tracts by Russell Howard and Victor Bonney respectively.

The whole volume is very readable, and is interestingly written. Printing and illustrations are clear, and the indexing is thorough. It can be safely recommended as a good and reliable exposition of modern, up-to-date surgery.

